# Efficient synthesis of chloro-derivatives of sialosyllactosylceramide, and their enhanced inhibitory effect on epidermal growth factor receptor activation

**DOI:** 10.3892/ol.2014.1887

**Published:** 2014-02-17

**Authors:** NAGAKO KAWASHIMA, HUANHUAN QU, MARLIN LOBATON, ZHENYUAN ZHU, MATTHIEU SOLLOGOUB, WEBSTER K. CAVENEE, KAZUKO HANDA, SEN-ITIROH HAKOMORI, YONGMIN ZHANG

**Affiliations:** 1 Division of Biomembrane Research, Pacific Northwest Research Institute, Seattle, WA 98122, USA; 2 Institute of Paris Molecular Chemistry, University Pierre & Marie Curie Paris 6, Paris 75005, France; 3 Glycochemistry and Glycobiology Lab, Shanghai Institute of Materia Medica, Chinese Academy of Sciences, Pudong, Shanghai 201203, P.R. China; 4 Ludwig Institute for Cancer Research, University of California, San Diego, La Jolla, CA 92093, USA; 5 Departments of Pathobiology and Global Health, University of Washington, Seattle, WA 98195, USA; 6 Institute for Interdisciplinary Research, Jianghan University, Wuhan Economic and Technological Development Zone, Wuhan, Hubei 430056, P.R. China

**Keywords:** glycosphingolipids, GM3, chloro-derivatives of GM3, EGFR activation, cell proliferation, EGFR inhibitors

## Abstract

Glycosphingolipids are components of essentially all mammalian cell membranes and are involved in a variety of significant cellular functions, including proliferation, adhesion, motility and differentiation. Sialosyllactosylceramide (GM3) is known to inhibit the activation of epidermal growth factor receptor (EGFR). In the present study, an efficient method for the total chemical synthesis of monochloro- and dichloro-derivatives of the sialosyl residue of GM3 was developed. The structures of the synthesized compounds were fully characterized by high-resolution mass spectrometry and nuclear magnetic resonance. In analyses of EGFR autophosphorylation and cell proliferation ([^3^H]-thymidine incorporation) in human epidermoid carcinoma A431 cells, two chloro-derivatives exhibited stronger inhibitory effects than GM3 on EGFR activity. Monochloro-GM3, but not GM3 or dichloro-GM3, showed a significant inhibitory effect on ΔEGFR, a splicing variant of EGFR that lacks exons 2–7 and is often found in human glioblastomas. The chemical synthesis of other GM3 derivatives using approaches similar to those described in the present study, has the potential to create more potent EGFR inhibitors to block cell growth or motility of a variety of types of cancer that express either wild-type EGFR or ΔEGFR.

## Introduction

Glycosyl structures, expressed as either glycoproteins or glycosphingolipids (GSLs), are involved in a variety of cell functions. Gangliosides (GSLs containing sialic acid) are abundantly expressed at the membranes of mammalian cells, particularly neuronal cells. The expression profiles of gangliosides and other GSLs have been shown to change during cell differentiation, proliferation and oncogenic transformation (1). The ganglioside sialosyllactosylceramide (GM3; NeuAcα3Galβ4Glcβ1Cer) inhibits the activity of various growth factor receptor (GFR)-associated tyrosine kinases. For example, the exogenous addition of GM3 has been shown to inhibit BHK cell growth induced by fibroblast growth factor (2) and the phosphorylation of platelet-derived GFR (3) and epidermal GFR (EGFR) (4). EGF-induced EGFR activation in human epidermoid carcinoma A431 cells was shown to be strongly inhibited by GM3, but to a much lesser degree by various other gangliosides and neutral GSLs. The order of inhibition was GM3>>GM2, GD3, GM4>GM1, GD1a, GD1b, GT1b, GD2, GQ1b>lactosyl-Cer (5). The inhibition of cell proliferation by exogenously added GM3 has also been reported (6,7).

In our previous preliminary study, it was found that fully halogenated GM3, starting from *N*-glycolyl-GM3, but not from *N*-acetyl-GM3, enhanced contact inhibition of tumor cell growth (8), indicating the possibility that the inhibitory effect of halogenated GM3 derivatives on EGFR activation is stronger than that of GM3 itself. The present study describes: (i) The complete chemical synthesis of monochloro-acetyl-GM3 and dichloro-acetyl-GM3 (referred to hereafter as monochloro-GM3 and dichloro-GM3, respectively), and (ii) evidence that these derivatives show stronger inhibitory effects in comparison with GM3, on the activation of EGFR and of ΔEGFR, a common mutant detected in cancers (9,10). The findings of the present study indicate the potential application of halogenated GM3 derivatives as a novel approach for cancer therapy.

## Materials and methods

### Synthesis of GM3 chloro-derivatives

All chemicals were reagent grade and used without further purification. Solvent ratios are by volume.

### Dichloromethane (CH_2_Cl_2_) was freshly distilled from P_2_O_5_

GM3 was synthesized as described previously (11) at the Institute of Paris Molecular Chemistry (University Pierre & Marie Curie Paris 6, Paris, France). Nuclear magnetic resonance (NMR) spectra were recorded with a Bruker DRX 400 spectrometer (400 MHz for ^1^H NMR and 100 MHz for ^13^C NMR; Bruker, Fällanden, Switzerland). The chemical shifts were referenced to the solvent peaks; δ=3.31 ppm (^1^H) and δ=49.00 ppm (^13^C) for CD_3_OD. The coupling constants were provided in Hz. High-resolution mass spectra (HRMS) were recorded with a Bruker micrOTOF spectrometer in electrospray ionization (ESI) mode, using the Tuning-Mix as a reference (Bruker). Reactions were monitored by thin-layer chromatography on glass plates precoated with silica gel 60 F_254_ (Merck, Darmstadt, Germany) and detected by charring with sulfuric acid. Flash column chromatography was performed on silica gel 60 (230–400 mesh; Merck).

**Figure 1:**
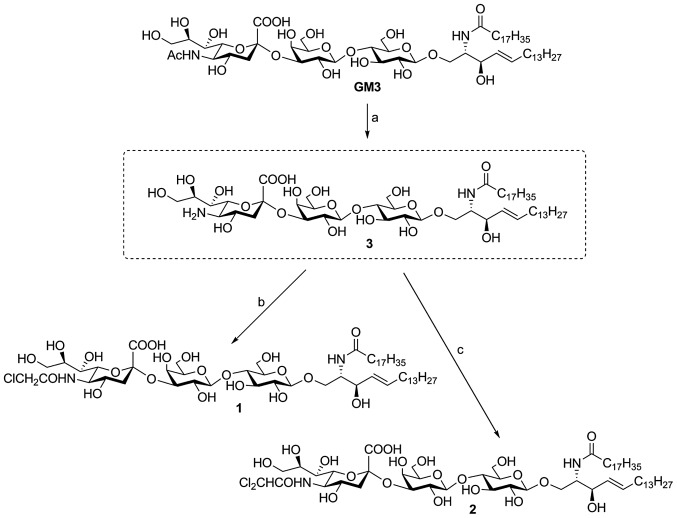
Method for synthesis of GM3 chloro-derivatives. Reagents and conditions: (A) 0.1 M KOH, 80°C, 5 h; (B) MeOH, CH_2_Cl_2_, Et_3_N, ClCH_2_COCl, r.t., 2 h, 49% (two steps from GM3); (C) MeOH, CH_2_Cl_2_, Et_3_N, Cl_2_CHCOCl, r.t., 2 h, 26% (two steps from GM3). Compound 1, monochloro-GM3; and compound 2, dichloro-GM3. GM3, sialosyllactosylceramide.

GM3 (30 mg, 0.025 mmol) in 10 ml 0.1 M KOH in H_2_O/butanol (1:9) solution was stirred at 80°C for 5 h. The mixture was neutralized by 6 M HCl (12–14) and concentrated *in vacuo*. The resulting residue was purified by flash column chromatography (CHCl_3_/MeOH, 2:1) to yield crude intermediate 3 (Fig. 1). To a solution of crude intermediate 3 (in 3 ml MeOH and 3 ml CH_2_Cl_2_), Et_3_N (74 μl, 0.53 mmol) and chloroacetyl chloride (40 μl, 0.50 mmol) were added. The mixture was stirred at room temperature (r.t). for 2 h and concentrated *in vacuo*. The resulting residue was purified by flash column chromatography (CHCl_3_/MeOH, 3:1) to produce compound 1. Compound 2 was prepared by the same procedure, except that dichloroacetyl chloride (48 μl, 0.50 mmol) was used instead of chloroacetyl chloride.

### Cell lines and culture

The human ovarian epidermoid cancer A431 cells were purchased from the American Type Culture Collection (Rockville, MD, USA). The human glioblastoma U87MG cell line and its stable transfectants, expressing wild-type EGFR (U87MG.wtEGFR) or mutant ΔEGFR (U87MG.ΔEGFR) (15,16), were from the Cavanee laboratory, Ludwig Institute for Cancer Research (San Diego, USA). Cells were grown in Dulbecco’s modified Eagle’s medium (DMEM) containing 10% heat-inactivated fetal bovine serum, 100 U/ml penicillin and 100 μg/ml streptomycin in 5% CO_2_ at 37°C in a humidified atmosphere.

### Reagents and antibodies

The GM3 used for the biological analyses was purchased from Matreya Inc., (Pleasant Gap, PA, USA) and dissolved in chloroform/methanol (C/M; 2:1) to make a stock solution (1 mg/ml). The antibodies used for western blotting were rabbit anti-EGFR monoclonal antibody (mAb; sc-03; Santa Cruz Biotechnology, Inc., Santa Cruz, CA, USA), rabbit anti-phospho-EGFR (PY1068) mAb (Epitomics, Burlingame, CA, USA) and mouse anti-GAPDH mAb (Millipore, Billerica, MA, USA).

### EGFR activation assay

EGFR autophosphorylation and the effects of GM3 and the chloro-derivatives were analyzed as described previously (17). GM3, dichloro-GM3 and monochloro-GM3 in C/M (2:1) were dried completely under N_2_ stream. The dried gangliosides were added with serum-free DMEM and sonicated for 10 min. The A431, U87MG.wtEGFR and U87MG.ΔEGFR cells were cultured in 24-well plates until ~90% confluency and starved in serum-free DMEM for 24 h. The starved cells were incubated in serum-free DMEM containing GM3, dichloro-GM3 or monochloro-GM3 for 16 h at 37°C. EGF (100 or 1 ng/ml) was added to the culture media, and the cells were further incubated for 30 min at 37°C. The cells were washed with Dulbecco’s phosphate-buffered saline (PBS) containing 500 nM Na_3_VO_4_, 5 mM EDTA, 5 mM NaF and 10 mM Na_4_O_7_P_2_ and then lysed with 100 μL radioimmunoprecipitation assay lysis buffer [1% Nonidet P-40, 25 mM Tris-HCl (pH 7.6), 150 mM NaCl, 1% deoxycholic acid and 0.1% SDS] containing 1% aprotinin, 1% phenylmethanesulfonyl fluoride, 500 nM Na_3_VO_4_ and Halt Protease and Phosphatase Inhibitor cocktail (Thermo Fisher Scientific, Inc., Waltham, MA, USA) for 30 min at 4°C. The solutions were centrifuged at 13,225 × g for 10 min at 4°C, and the supernatants were collected and used as cell lysates. The protein concentration of the cell lysates was determined using a Micro Bicinchoninic Acid Protein Assay kit (Pierce Biotechnology, Inc., Rockford, IL, USA).

### SDS-PAGE and western blot analysis

The cell lysates were analyzed by western blotting, as described previously (18–21). In brief, subsequent to boiling for 5 min at 98°C in SDS sample buffer, 5 μg protein of each cell lysate was resolved by 7.5% SDS-PAGE and transferred onto polyvinylidene fluoride membranes (Thermo Fisher Scientific, Inc.). The membranes were blocked with 3% bovine serum albumin/Tris-buffered saline (TBS) containing 0.1% Tween-20 (TBS-T) for 1 h at r.t., incubated with specific primary antibodies in TBS-T for 2 h at r.t. or overnight at 4°C and washed. The membranes were then incubated with appropriate secondary antibodies conjugated with horseradish peroxidase in TBS-T for 1 h at r.t. and washed 3 times with TBS-T. Detection was performed by enhanced chemiluminescence using SuperSignal West Pico chemiluminescent substrate (Thermo Fisher Scientific, Inc.). The intensity of western blotting was determined by densitometry using the ImageJ program (http://rsb.info.nih.gov/ij/).

### [^3^H]-thymidine cell incorporation assay

[^3^H]-thymidine incorporation into DNA was used as a measure of the DNA replication level, following the method of Gabelman and Emerman (22). The A431, U87MG.wtEGFR and U87MG.ΔEGFR cells were cultured in 48-well plates in DMEM containing 10% fetal bovine serum until 70–80% confluence, and then starved in serum-free DMEM for 24 h. The starved cells were incubated in serum-free DMEM containing GM3, monochloro-GM3 or dichloro-GM3 for 16 h at 37°C. EGF (100 or 1 ng/ml) was added to the culture media and the cells were further incubated for 2 or 24 h at 37°C. The cells were then incubated with 0.8 μCi of [^3^H]-thymidine (PerkinElmer, Waltham, MA, USA) for 4 h at 37°C, washed 3 times with PBS and detached with trypsin/EDTA. The cell suspension was mixed with Ecoscint (1:30; National Diagnostics, Atlanta, GA, USA), and [^3^H]-thymidine incorporation was determined by a liquid scintillation β-counter (Beckman Instruments, Fullerton, CA, USA).

### Statistical analysis

The data were analyzed by Student’s t-test. P≤0.05 was considered to indicate a statistically significant difference.

## Results

### Synthesis of GM3 chloro-derivatives

GM3 was synthesized, as described previously (11), at the Institute of Paris Molecular Chemistry (University Pierre & Marie Curie Paris 6, Paris, France). A suitably protected lactoside diol was glycosylated with sialyl xanthate to exclusively produce the α-sialyl trisaccharide at a good yield based on a highly stereoselective and regioselective sialylation. Following chemical modification, this trisaccharide was reacted with 3-O-benzoylated azidosphingosine to form a GSL, which subsequent to a reduction of azide followed by condensation with stearic acid and deprotection, yielded GM3.

Under strongly basic conditions (0.1 M KOH, 80°C), the *N*-acetyl group of GM3 was hydrolyzed to yield the key intermediate 3 (Fig. 1), in which the free amino functionality could be subjected to derivatization. Two *N*-modified GM3 analogues, compound 1 (monochloro-GM3) and compound 2 (dichloro-GM3) (Fig. 1), were synthesized from intermediate 3.

Compound 1 was obtained (14.9 mg, 49% for two steps) as a white foam with a retention factor (Rf) value of 0.45 (EtOAc-iPrOH-H_2_O, 3:2:1) and an [α]_D_ of −0.3 (c, 0.5; and CHCl_3_:MeOH, 1:1). The NMR spectral data were in good agreement with results reported previously (23). The ESI-HRMS (m/z) for C_59_H_106_ClN_2_O_21_ [M-H]^−^ was calculated as 1213.6982 m/z and found to be 1213.7015 m/z.

Compound 2 was obtained (8.1 mg, 26% for two steps) as a white foam, with an Rf of 0.53 (EtOAc-iPrOH-H_2_O, 3:2:1); and an [α]_D_ of −0.4 (c, 0.5; and CHCl_3_:MeOH, 1:1). ^1^H NMR (400 MHz; CDCl_3_:CD_3_OD, 1:1): δ 6.16 [singlet (s), 1H, Cl_2_CH], 5.63 [triple doublet (td), coupling constants (J)=15.0, 6.8 Hz, 1H, H-5cer], 5.38 [double doublet (dd), J=15.3, 7.6 Hz, 1H, H-4cer], 4.36 (d, J=7.8 Hz, 1H, H-1Gal), 4.24 (d, J=7.8 Hz, 1H, H-1Glu), 4.14 (dd, J=9.9, 4.0 Hz, 1H, Ha-1cer), 4.04 (dd, J=8.5, 5.9 Hz, 1H, H-3cer), 4.01-3.89 [multiplet (m), 3H, H-3Gal, H-2cer, H-4Gal], 3.89-3.78 (m, 4H, Ha-6Gal, Hb-6Gal, Ha-6Glu, H-6Neu), 3.77-3.68 (m, 4H, Ha-9Neu, H-4Neu, H-5Neu, H-5Gal), 3.66-3.46 (m, 7H, Hb-6Glu, H-5Glu, Hb-9Neu, H-8Neu, H-2Gal, H-3Glu, H-4Glu), 3.44 (d, J=9.0 Hz, 1H, Hb-1cer), 3.40-3.34 (m, 1H, H-7Neu), 3.24 (d, J=10.2 Hz, 1H, H-2Glu), 2.88-2.67 (m, 1H, Heq-3Neu), 2.11 [triplet (t), J=7.6 Hz, 2H, CH_2_C(O)], 1.96 (dd, J=12.7, 5.8 Hz, 2H, H2-6cer), 1.76-1.66 (m, 1H, Hax-3Neu), 1.56-1.48 (m, 2H, CH_2_CH_2_C(O)), 1.25-1.19 (m, 50H, alkane CH_2_) and 0.82 (t, J=6.8 Hz, 6H, 2xCH_3_). ^13^C NMR (100 MHz; CDCl_3_:CD_3_OD, 1:1): δ 134.92 (C-5cer), 130.25 (C-4cer), 104.48 (C-1Gal), 103.73 (C-1Glu), 80.36, 76.95, 76.21, 75.66, 75.39, 74.05, 73.95, 72.51, 72.37, 70.10, 69.42 (C-1cer), 68.43, 68.31, 66.94 (Cl_2_CH), 64.05, 63.91, 62.25, 61.18, 53.99 (C-2cer), 53.74 (C-5Neu), 41.30 (C-3cer), 37.04, 33.01, 32.53, 30.31, 30.24, 30.22, 30.12, 29.97, 29.91, 26.64, 23.24 and 14.34 (2xCH_3_). ESI-HRMS (m/z) for C_59_H_105_Cl_2_N_2_O_21_ [M-H]^−^ was calculated as 1247.6592 m/z and found to be 1247.6595 m/z.

### Inhibitory effect of GM3 chloro-derivatives on EGFR activation

**Figure 2:**
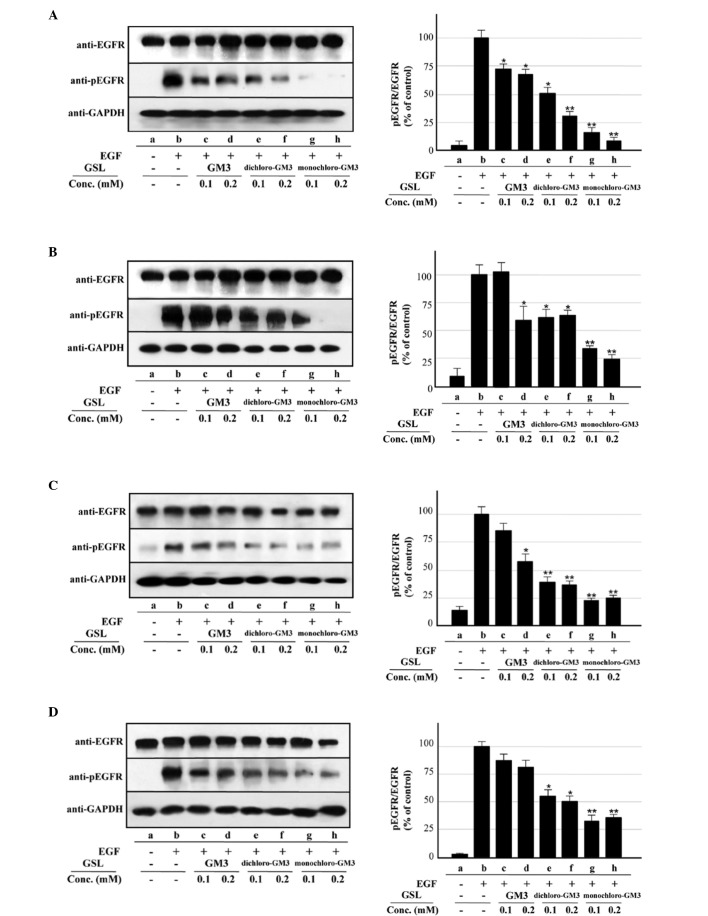

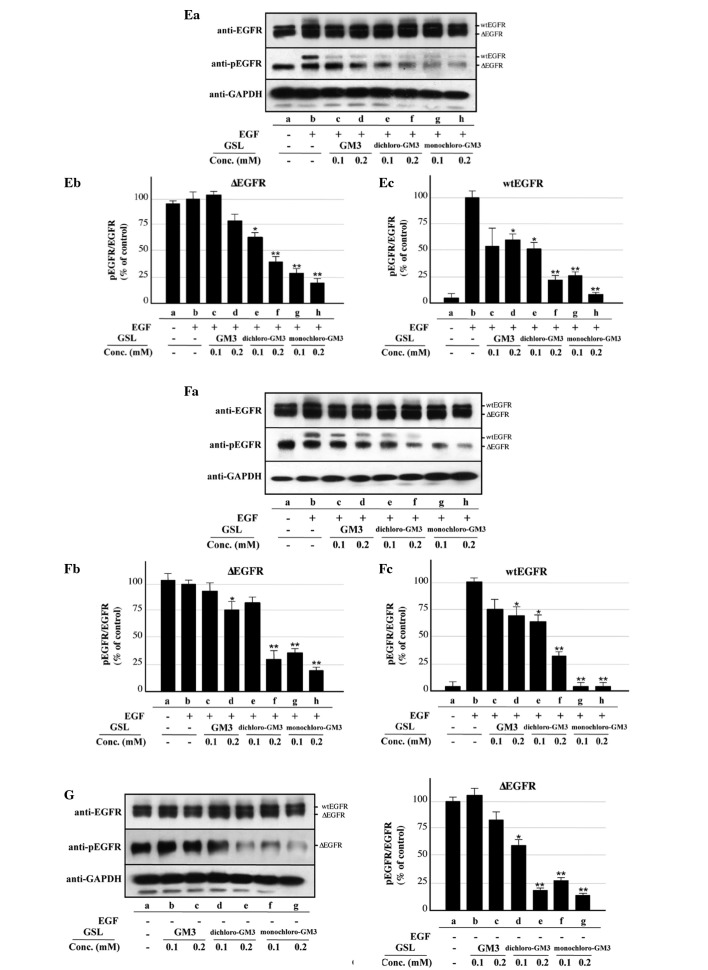
Inhibitory effects of pre-incubation with GM3, monochloro-GM3 and dichloro-GM3 on EGFR activation (autophosphorylation) in the A431, U87MG.wtEGFR and U87MG.ΔEGFR cells. (A) A431 cells, 100 ng/ml EGF. (B) A431 cells, 1 ng/ml EGF. (C) U87MG.wtEGFR cells, 100 ng/ml EGF. (D) U87MG.wtEGFR cells, 1 ng/ml EGF. Left, representative western blotting results from triplicate experiments. GAPDH signals are shown to confirm the protein amounts in the cell lysates. Right, the y-axis indicates the ratio of phosphorylated EGFR (pEGFR) to EGFR as a percentage of the control value. The control is b in panels A–F and a in panel G. (E) U87MG.ΔEGFR cells, 100 ng/ml EGF. (F) U87MG.ΔEGFR cells, 1 ng/ml EGF. (G) U87MG.ΔEGFR cells, no EGF. The results are presented as the mean ± dtandard deviation. ^*^P<0.05; ^**^P<0.01. GM3, sialosyllactosylceramide; EGFR, epidermal growth factor receptor; GSL, glycosphingolipids.

The inhibitory effects of monochloro- and dichloro-GM3 on EGF-induced EGFR activation were evaluated and compared with that of GM3 using A431 cells, which express a high level of EGFR at the cell surface. In agreement with the findings of our previous studies (3,4,18,19,24), pre-incubation of the cells with GM3 at either 0.1 or 0.2 mM caused ~65% inhibition of EGFR activation (as assessed by its autophosphorylation) induced by 100 ng/ml EGF (Fig. 2A, columns c and d). The degree of inhibition by the chloro-derivatives at concentrations of 0.1 and 0.2 mM was ~50 and ~70% for dichloro-GM3 (Fig. 2A, columns e and f) and ~85 and ~90% for monochloro-GM3 (Fig. 2A, columns g and h), respectively. For EGFR activation induced by 1 ng/ml EGF, 0.2 mM GM3 caused ~40% inhibition and 0.1 mM GM3 exhibited no significant effect (Fig. 2B, columns c and d). The degree of inhibition at concentrations of 0.1 and 0.2 mM was ~37 and ~32% for dichloro-GM3 (Fig. 2B, columns e and f) and ~65 and ~75% for monochloro-GM3 (Fig. 2B, columns g and h), respectively. The inhibitory effects of GM3 and the chloro-derivatives on EGFR activation in the U87MG.wtEGFR cells were quite similar for EGF concentrations of 100 (Fig. 2C, columns c–h) and 1 ng/ml (Fig. 2D, columns c–h).

The inhibitory effects of GM3 and its chloro-derivatives were also evaluated in U87MG.ΔEGFR cells, which overexpress the highly oncogenic mutant ΔEGFR gene, a commonly occurring variant of EGFR that lacks exons 2–7 (25,26). These exons code for the N-terminal region that includes the EGF binding site, and the deleted form of EGFR is therefore constitutively autophosphorylated without EGF stimulation in U87MG.ΔEGFR cells (15,16). In the present study, two types of EGFR (endogenous wtEGFR and transfected ΔEGFR) were detected in these cells as expected, and ΔEGFR autophosphorylation was detected at similar levels regardless of the presence or absence of EGF. In contrast to the results for wtEGFR, ΔEGFR autophosphorylation was not significantly inhibited by 0.1 mM GM3, but was ~25% inhibited by 0.2 mM GM3 at 100 (Fig. 2Eb, columns b–d) and 1 ng/ml EGF (Fig. 2Fb, columns b–d). The chloro-derivatives showed notable inhibitory effects under all three conditions: 100 ng/ml EGF (Fig. 2E), 1 ng/ml EGF (Fig. 2F) and no EGF (Fig. 2G). Monochloro-GM3 produced ~73 and ~80% inhibition at concentrations of 0.1 and 0.2 mM, respectively (Fig. 2Eb and Fb, columns b, g and h; Fig. 2G, columns a, f and g). The inhibitory effects of dichloro-GM3 were stronger than those of GM3, but less than those of monochloro-GM3 (Fig. 2E–G). The inhibitory effects of GM3 and the chloro-derivatives on endogenous wtEGFR expressed in the U87MG.ΔEGFR cells were extremely similar to those observed for the A431 and U87MG.wtEGFR cells (Fig. 2Ec and Fc).

### Inhibitory effects of GM3 and its chloro-derivatives on cell proliferation

**Figure 3:**
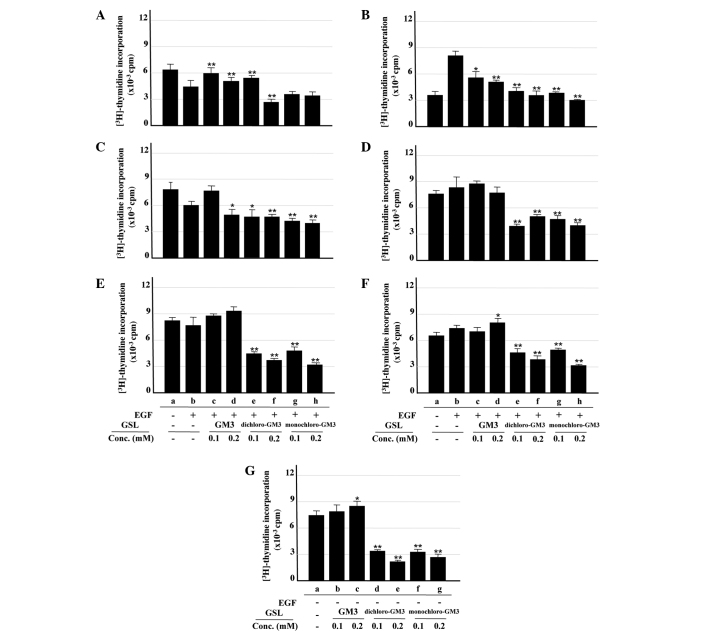
Inhibitory effects of GM3, monochloro-GM3 and dichloro-GM3 on cell proliferation, assessed by [^3^H]-thymidine incorporation assay. (A) A431 cells, 100 ng/ml EGF. (B) A431 cells, 1 ng/ml EGF. (C) U87MG.wtEGFR cells, 100 ng/ml EGF. (D) U87MG.wtEGFR cells, 1 ng/ml EGF. (E) U87MG.ΔEGFR cells, 100 ng/ml EGF. (F) U87MG.ΔEGFR cells, 1 ng/ml EGF. (G) U87MG.ΔEGFR cells, no EGF. [^3^H]-thymidine incorporation was expressed as cpm. All experiments were performed in triplicate and results are presented as the mean ± standard deviation. The control is b in panels A–F and a in panel G. ^*^P<0.05; ^**^P<0.01. GM3, sialosyllactosylceramide; EGF, epidermal growth factor; GSL, glycosphingolipids; cpm, counts per minute.

The inhibitory effects of GM3 and its chloro-derivatives on cell proliteration were analyzed by the [^3^H]-thymidine incorporation assay. Treatment with EGF at 100 ng/ml, the concentration used for analysis of EGFR activation in the A431 cells, inhibited rather than promoted cell proliferation (data not shown). As indicated in our previous study (24), this result may be due to the excessive expression of EGFR on the surface of the A431 cells. As 1 ng/ml EGF induced cell proliferation (Fig. 3B, columns a and b), this concentration was used for the analysis of the inhibitory effects of GM3 and the chloro-derivatives. The chloro-derivatives, particularly monochloro-GM3, significantly inhibited [^3^H]-thymidine incorporation into the A431 cells (Fig. 3A and B, columns b, e–h), whereas GM3 showed an enhancing effect (Fig. 3A and B, columns b–d). The mechanism of this enhancing effect remains unclear, but it may be associated with the excessive expression of EGFR on the surface of the A431 cells, as aforementioned. Similar inhibitory effects of the chloro-derivatives were observed in the U87MG.wtEGFR cells at the EGF concentrations used (Fig. 3C and D, columns a–h). The chloro-derivatives significantly inhibited [^3^H]-thymidine incorporation into the U87MG.ΔEGFR cells in the presence and absence of EGF, whereas GM3 exhibited no significant inhibitory effect (Fig. 3E and F, columns a–h; Fig. 3G, columns a–g). These findings were consistent with those from the analysis of ΔEGFR autophosphorylation, as described in the preceding section.

## Discussion

The expression of glycosyl epitopes, carried as glycoproteins or GSLs on the surface of mammalian cells, is known to vary quantitatively and qualitatively in association with various cell phenotypes. GM3, a sialic acid-containing GSL whose expression is reduced in transformed cells, inhibits EGFR activation, a process associated with cancer cell growth and motility (4).

Studies utilizing synthetic molecules that resemble natural GSLs provide important information that is not available from studies utilizing molecules from natural sources. In the present study, to elucidate the inhibitory effects of the chloro-derivatives of GM3 on EGFR activation, a simple and efficient synthetic route was developed for the preparation of monochloro- and dichloro-GM3. The key step is a highly regioselective and stereoselective sialylation from a suitably protected lactoside diol with a sialyl xanthate, to exclusively provide the α-sialyl trisaccharide at a good yield. Selective hydrolysis of the *N*-acetyl group of GM3 was achieved under basic conditions (0.1 M KOH, 80°C) to yield key intermediate 3 (Fig. 1), from which the two chloro-derivatives were prepared.

The finding that two halogen-(chloro-) derivatives of GM3 have stronger inhibitory effects on EGFR activation than GM3, indicates that the chemical synthesis approach described in the present study can be applied for the development of more potent inhibitors. The effects of trichloro-derivatives and fluoro-derivatives of GM3 should also be evaluated. It is noteworthy that monochloro-GM3 inhibited the activation of ΔEGFR, which is commonly expressed in glioblastomas (the most aggressive type of brain tumor in humans) and whose continuous EGF-independent activation is associated with greatly enhanced malignant behavior. The inhibitory effects of the two chloro-derivatives on EGFR activation (autophosphorylation) and cell proliferation ([^3^H]-thymidine incorporation) were stronger than that of GM3. Monochloro-GM3 also exhibited a significant inhibitory effect on ΔEGFR, which is a mutant form of EGFR often found in glioblastomas.

Our previous studies have shown that: (i) GM3 inhibits EGFR activation through carbohydrate-to-carbohydrate interaction; (ii) GM3 interacts with *N*-linked glycans carrying multiple GlcNAc termini carried by EGFR; and (iii) such interaction is the molecular mechanism whereby GM3 inhibits EGFR activation (18,19). Studies are in progress to determine whether monochloro- and dichloro-GM3 bind more strongly than GM3 to *N*-linked glycans carrying multiple GlcNAc termini.

The chemical synthesis of other GM3 derivatives using approaches similar to that described in the present study has the potential to create more potent EGFR inhibitors.

## Abbreviations

DMEM: Dulbecco’s modified Eagle’s medium
GSL: glycosphingolipid
EGF: epidermal growth factor
EGFR: epidermal growth factor receptor
GM3: sialosyllactosylceramide
HRMS: high-resolution mass spectrometry
NMR: nuclear magnetic resonance
PBS: phosphate-buffered saline
r.t.: room temperature
TBS: Tris-buffered saline
